# N^10^‐carbonyl‐substituted phenothiazines inhibiting lipid peroxidation and associated nitric oxide consumption powerfully protect brain tissue against oxidative stress

**DOI:** 10.1111/cbdd.13572

**Published:** 2019-06-12

**Authors:** Robert G. Keynes, Anastasia Karchevskaya, Dieter Riddall, Charmaine H. Griffiths, Tomas C. Bellamy, A. W. Edith Chan, David L. Selwood, John Garthwaite

**Affiliations:** ^1^ Neural Signalling Group The Wolfson Institute for Biomedical Research University College London London UK; ^2^ Drug Discovery Group The Wolfson Institute for Biomedical Research University College London London UK

**Keywords:** antioxidant, ferroptosis, lipid peroxidation, neurodegeneration, nitric oxide, phenothiazine, XED

## Abstract

During some investigations into the mechanism of nitric oxide consumption by brain preparations, several potent inhibitors of this process were identified. Subsequent tests revealed the compounds act by inhibiting lipid peroxidation, a trigger for a form of regulated cell death known as ferroptosis. A quantitative structure–activity study together with XED (eXtended Electron Distributions) field analysis allowed a qualitative understanding of the structure–activity relationships. A representative compound N‐(3,5‐dimethyl‐4H‐1,2,4‐triazol‐4‐yl)‐10H‐phenothiazine‐10‐carboxamide (DT‐PTZ‐C) was able to inhibit completely oxidative damage brought about by two different procedures in organotypic hippocampal slice cultures, displaying a 30‐ to 100‐fold higher potency than the standard vitamin E analogue, Trolox or edaravone. The compounds are novel, small, drug‐like molecules of potential therapeutic use in neurodegenerative disorders and other conditions associated with oxidative stress.

## Graphical abstract

N^10^‐carbonyl‐substituted phenothiazines exemplified by DT‐PTZ‐C inhibit lipid peroxidation and oxidative stress, which are key drivers of ferroptosis, a form of regulated cell death.

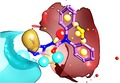

## 1 INTRODUCTION

Lipid peroxidation is a key factor in numerous disease states where oxidative stress has been implicated, including neurodegenerative disorders (Hambright, Fonseca, Chen, Na, & Ran, 2017) such as motor neuron disease (Cacabelos et al., 2014) and multiple sclerosis (Hu et al., 2018). Other conditions such as cardiovascular disease, asthma and diabetes may also involve oxidative stress. (Ozbayer et al., 2018). Severe lipid peroxidation triggers a form of regulated cell death that is initiated by oxidative perturbations of the intracellular microenvironment and that can be inhibited by iron chelators and lipophilic antioxidants (Galluzzi et al., 2018; Gaschler & Stockwell, 2017). Originally termed oxytosis (Tan, Schubert, & Maher, 2001), this has been shown to be identical to the pathway termed ferroptosis (Lewerenz, Ates, Methner, Conrad, & Maher, 2018). The reduced glutathione (GSH)‐dependent enzyme glutathione peroxidase 4 (GPX4), by catalyzing the reduction of lipid peroxides to alcohols, is considered a key defense against oxytosis/ferroptosis (Friedmann Angeli et al., 2014). Depletion of GSH may also initiate oxytosis/ferroptosis, a process implicated in the cellular toxicity of glutamate mediated by inhibition of cystine uptake (Dixon et al., 2012). The brain is thought to be particularly susceptible to oxidative stress because of its high unsaturated lipid content and mitochondrial activity, among other factors (Cobley, Fiorello, & Bailey, 2018).

Lipid peroxidation is initiated by radical species such as the hydroxyl radical (^**•**^OH), which can abstract a hydrogen atom from unsaturated lipid, thus generating a lipid radical (L^**•**^) and H_2_O. The lipid radical can combine with O_2_, generating the lipid “peroxyl” radical (LOO^**•**^), which can further react with unsaturated lipid. If allowed to progress unchecked, a damaging, self‐propagating cascade of peroxidation results. Ultimately, peroxidation alters membrane properties, including ion‐channel activity and glucose transport, and can directly impair mitochondrial function to cause cell stress (Mattson, 1998).

The ^**•**^OH usually thought to initiate lipid peroxidation (Koppenol, 2001) can be formed by either the Haber–Weiss reaction (1), though this is probably too slow at neutral pH, or more rapidly by the transition metal‐catalyzed Fenton reaction (2):

(1) $${\text{O}}_{2}^{\cdot -}+{\text{H}}_{2}{\text{O}}_{2}\to {\text{O}}_{2}+{\text{OH}}^{-}+{}^{\cdot }\text{OH}$$

(2) $${\text{Fe}}^{2+}/{\text{Cu}}^{+}+{\text{H}}_{2}{\text{O}}_{2}\to {\text{Fe}}^{3+}/{\text{Cu}}^{2+}+{\text{OH}}^{-}+{}^{\cdot }\text{OH}$$

We recently found that freshly isolated cell suspensions or homogenates from rat brain consume NO in a lipid peroxidation‐dependent manner. Untreated, these preparations undergo spontaneous and continuous lipid peroxidation as they contain suitable concentrations of iron and ascorbate to initiate this reaction. NO consumption by this mechanism may be prevented by treatment with metal ion chelators (DTPA, EGTA), by ascorbate depletion (ascorbate oxidase), or by treatment with antioxidant compounds (Trolox and the lazaroid U‐74389G; Keynes, Griffiths, Hall, & Garthwaite, 2005). Physiologically, other mechanisms of NO consumption are likely to dominate in the intact brain (Hall & Garthwaite, 2006), although the lipid peroxidation‐dependent process may become important under pathological conditions.

**Figure 1:**
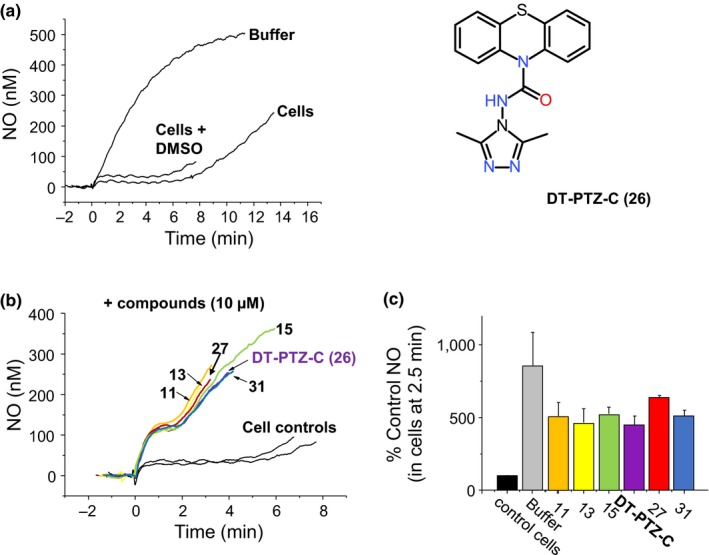
Representative traces (a) and (b) and summary of the data (c) of NO consumption after addition of the NO donor DETA/NO (200 μM) to buffer or cerebellar cells (20 × 10^6^ cells/ml; 1.25 mg protein/ml) in the absence or presence of compound numbers **26**,** 27**,** 11**,** 19**,** 15**, and **91**. Data are *n* = 2 ± *SD*

We report here the identification of a novel series of small drug‐like molecules based on a phenothiazine core that inhibit NO consumption and show that they do so by preventing lipid peroxidation with a high potency. Although phenothiazines are well known as antioxidants, our unbiased screening method identified a subset of molecules with high potency that could not have been predicted from previous studies. An exemplar molecule, N‐(3‐methyl‐4H‐1,2,4‐triazol‐4‐yl)‐10H‐phenothiazine‐10‐carboxamide, DT‐PTZ‐C, (Figure 1) compound **26**, showed high potency and efficacy in protection of hippocampal slice cultures.

The development of drugs with an antioxidant mechanism of action has not been straightforward with many failures reported (Steinhubl, 2008),(Ohlow, Sohre, Granold, Schreckenberger, & Moosmann, 2017). The field has recently been given fresh impetus by the demonstration of clinical efficacy for edaravone in motor neuron disease and its approval in Japan and in the United States (Abe et al., 2017). The development of new and more efficacious therapies is therefore of interest.

## 2 METHODS

### 2.1 Compound sourcing, similarity, and substructure searching

The selection of compounds for testing consisted of two steps. The first search was performed using a 2D fingerprint MACCS keys (Durant, Leland, Henry, & Nourse, 2002) similarity search against the ACD (Willett, Barnard, & Downs, 1998). The Tanimoto coefficient (Tanimoto, 1958) was set at 0.75. Once the phenothiazine was identified as a core scaffold, this was used to further reduce the number of initial hits by substructure search. Compounds were sourced from diverse commercial suppliers and checked by LCMS for purity with a threshold of 95%. DT‐PTZ‐C is available from multiple chemical suppliers (Supplementary information).

### 2.2 Molecular modeling and QSAR

The MOE modeling package was used. Molecules were drawn using ISIS draw or Chemdraw and imported into MOE using sdf. All the available IC_50_ data were used (26 compounds) in the analysis. The objective was to obtain a good QSAR model representing the data, not to predict the activity of unknown compounds. In this case, training and test set protocols were not utilized.

### 2.3 XED force field analysis

The 3D‐QSAR software Cresset Forge V. 10. was used following the default procedures (Cheeseright, Mackey, Rose, & Vinter, 2006). Cresset uses a field point description of the molecules (Tedesco). This is based on (a) a field, which combines the electrostatic parameters (positive and negative fields) of the molecule(s) and the surface interaction field (Cheeseright et al.,^2006^), and (b) the points, which are the extreme values of the field (Cheeseright et al., 2006). A probe is required for the comparison, so the field values can be determined. The field value consists of the interaction energy and the charged probe at the center of the estimated point. The surface energy is represented by the van der Waals energy and the neutral probe (Cheeseright et al., 2006).

### 2.4 Brain homogenate preparation

Whole brain homogenate (~20 mg protein/ml) was prepared from 8‐day‐old Sprague‐Dawley rats by sonication in 20 mM tris buffer (pH 7.4). The homogenate was either stored at −20°C until use or was further fractionated by centrifugation at 4°C. After an initial spin (10,000 *g* for 30 min), the pellet was discarded and the supernatant further spun at 100,000 *g* for 1 hr. The resultant pellet was resuspended in tris buffer (20 mM) at 10 mg protein/ml while the supernatant was spun overnight at 2,000 *g* through 10,000 kDa cutoff filters (CENTRIPLUS^®^, Millipore UK Ltd). This procedure was carried out to remove free hemoglobin without compromising NO consumption on recombination with the pellet. The 100,000 *g* pellet and filtered supernatant were stored at −20°C until use.

### 2.5 NO consumption assay

A modification of the standard oxyhemoglobin assay (Livingston, 1996) was used to monitor NO consumption by brain preparations and subsequently detect inhibitors of this activity. Hemoglobin‐coated beads (12–16 mg/ml) were triple washed in tris buffer (20 mM) before exposure to freshly prepared sodium dithionate (10 mM) for 20 min in air to reduce the hemoglobin to the ferrous (Fe^2+^) form. Following a further two washes in tris, the beads were kept as a working stock at 1.2 mg/ml on ice until used. Brain pellet (0.1 mg/ml), supernatant (10%) or in later experiments ascorbate (30 μM), and superoxide dismutase (SOD, 1000 U/ml) were incubated with tris buffer and hemoglobin beads (100 μl), in a final volume of 1 ml on a rotator at 37°C for up to 25 min. Inhibitor test compounds were added where appropriate. All test compound stocks were prepared in DMSO. After incubation, the bead mix was pelleted by centrifugation at 10,000 *g* for 5 min and resuspended in 300 μl tris. The degree of hemoglobin oxidation was determined by reading the absorbance ratio (401–410 nm/410 nm).

### 2.6 Lipid peroxidation assay

The levels of thiobarbituric acid‐reactive species (TBARS) were determined using a published assay (Esterbauer & Cheeseman, 1990). The method is based on the reaction of lipid peroxidation breakdown products, mainly malondialdehyde, with thiobarbituric acid. A pink reaction product is produced, and the absorbance is read at 532 nm. This assay is reliably used for comparison of antioxidant compounds, for example in rat brain homogenates (Callaway, Beart, & Jarrott, 1998) or phospholipid vesicles (Westerlund, OstlundLindqvist, Sainsbury, Shertzer, & Sjoquist, 1996). It has been reported that the TBARS assay can give misleading results (Forman et al., 2015), but the version we used, where protein is precipitated and removed prior to reaction, has been shown to be reliable and to correlate closely with direct measurements of malondialdehyde, the main lipid peroxidation breakdown product, in brain preparations(Callaway et al., 1998). In addition, we show below that the results of the TBARS assay correlate closely with those for the NO consumption assay, which is the result expected if the two were independently measuring the degree of lipid peroxidation.

Inhibitor test compounds were incubated with brain pellet (0.1 mg/ml), supernatant (10%) or, in later experiments, ascorbate (30 μM), and SOD 1,000 U/ml in tris buffer (20 mM) in a final volume of 1 ml on a rotator at 37°C for up to 25 min. Samples were inactivated by addition of trichloroacetic acid (10% w/v) at 4°C and were centrifuged to remove precipitated protein (2,000 *g*, 10 min). The supernatant was added to a mixture of thiobarbituric acid (0.67% w/v) and butylated hydroxytoluene (10% w/v) and was then heated to 90 °C for 30 min. After cooling to room temperature, the absorbance of the solution was measured at 510 and 532 nm and the absorbance ratio (532–510 nm)/510 nm was calculated. The concentration was determined by reference to malondialdehyde standards.

### 2.7 Monitoring NO consumption in cerebellar cell suspensions

Acute cerebellar cell suspensions (20 × 10^6^ cells/ml; 1.25 mg protein/ml) were prepared from 8‐day‐old Sprague‐Dawley rats according to published procedures (Garthwaite & Garthwaite, 1987) except that the pups were not pre‐treated with hydroxyurea. The cell incubation medium contained (mM) NaCl (130), KCl (3), CaCl_2_ (1.5), MgSO_4_ (1.2), Na_2_HPO_4_ (1.2), tris‐HCl (15) and glucose (11) adjusted to pH 7.4 at 37°C.

NO consumption by the cell suspension was studied following the addition of the NO donor diethylenetriamine/NO adduct DETA/NO (200 μM; Alexis) which was made in 10 mM NaOH and kept on ice until use. For measurements of NO concentrations, 1 ml of samples was incubated in a stirred chamber (at 37°C) equipped with an NO electrode (ISO‐NO, World Precision Instruments) in the presence or absence of test compounds. All experiments contained superoxide dismutase (SOD, 1,000 U/ml). Other stock solutions (from Sigma) were made up at 1000 x concentration in DMSO so that the final DMSO concentration did not exceed 0.1%.

### 2.8 Hippocampal slice culture preparation

Slice cultures were prepared according to a standard method (Stoppini, Buchs, & Muller, 1991). Sprague‐Dawley rat brains were immersed in ice‐cold minimal essential medium supplemented with 10 mM tris, and penicillin/streptomycin (100 U/ml and 100 μg/ml, respectively). Hippocampi were rapidly dissected out, and 400 μm transverse sections prepared on a McIlwain tissue chopper (Mickle Laboratory Engineering Ltd). Slices were separated mechanically and randomized before being placed onto culture inserts (Millicell‐CM: Millipore, 4 slices per insert). Culture inserts were incubated in 6‐well plates with 1 ml media consisting of minimal essential medium (50%), heat‐inactivated horse serum (25%), Hank's balanced salt solution (25%), and penicillin/streptomycin (as above), buffered to pH 7.3 with tris (5 mM) and NaHCO_3_ (0.35 g/L). Cultures were incubated at 37°C in 5% CO_2_ for 4 days and subsequently at 33°C in 5% CO_2_ until use at 12–14 days in vitro. Inserts were transferred to fresh media after 1, 4, 7, and 10 days.

### 2.9 Slice culture toxicity model

Hippocampal slice cultures (12–14 days old) were incubated in serum‐free medium (SFM) consisting of minimal essential medium without HEPES (74%), Hank's balanced salt solution (24%), B27 supplement without antioxidants (2%), penicillin/streptomycin (as above), and glucose (0.5 g/L) for 2 hr before exposure to freshly prepared ascorbate (500 μM) and FeSO_4_ (10–1,000 μM). Alternatively, cultures were exposed to 2,2′‐azo‐bis‐amidinopropane (ABAP) (0.3–3 mM). Stock compounds Trolox and DT‐PTZ‐C were prepared at 1,000‐times final concentration in DMSO and were present in the SFM throughout the experiment when used. Neuronal damage was assessed by propidium iodide staining after 24 hr according to the procedure published previously (Keynes, Duport, & Garthwaite, 2004).

## 3 RESULTS

### 3.1 Identification of potent NO consumption inhibitors

A modified oxyhemoglobin assay was used to screen for compounds that inhibit NO consuming activity. The ability of test compounds to inhibit NO consumption was determined after 25 min and compared to the effect of the calcium chelator EGTA, which gave 100% inhibition (Keynes et al., 2005). The first two compounds tested, chosen on the basis of initial serendipitous observations, were calmodulin antagonists. Both compounds inhibited NO consumption with potencies similar to that of EGTA (Table 1).

**Table 1:** Calmodulin antagonists inhibit NO consumption with similar potency to EGTA

Compound	Structure	IC_50_ (μM)	Molecular weight	ClogP
EGTA	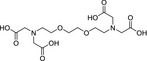	16	380.4	−2.0
Trifluoroperazine	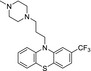	9	407.5	4.9
Calmidazolium	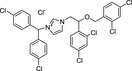	10	652.2	6.9

A further screening round was undertaken for **40** compounds available in the laboratory, of which 7 inhibited NO consumption with an IC_50_ under 100 μM (Table 2).

**Table 2:** Second round of screening for inhibitors of NO consumption

Compound	Structure	IC_50_ (μM)	Molecular weight	ClogP
Nifedipine	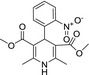	70	346.3	3.1
Clozapine	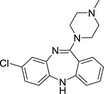	17	326.8	3.6
Desipramine	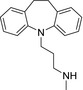	15	266.4	4.5
Bepridil	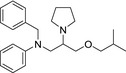	12	366.5	6.2
Chlorpromazine	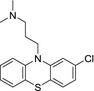	7	318.9	5.5
Promethazine	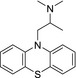	7	284.4	4.6
Methotrimeprazine	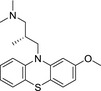	4	328.5	5.0

Finally, a set of compounds based upon the phenothiazine structure of the most potent inhibitors (chlorpromazine, promethazine, methotrimeprazine) were selected using similarity and substructure searching. The compounds were screened, and the summary data compare these compounds to control values (no inhibitor present) at the lowest concentration screened initially (0.4 or 1 μM). Of the **61** compounds tested, full IC_50_ values were subsequently determined for the best compounds (Table S1). The compounds are grouped into structural types N‐carbonylphenothiazines, N‐alkylphenothiazines and diverse structures and phenothiazines unsubstituted on the ring nitrogen (Table S1).

### 3.2 NO consumption inhibitors are active in an intact cell system

We have previously shown that intact cells isolated from the cerebellum consume NO by a mechanism at least partly explained by lipid peroxidation. This proportion of cellular NO consumption can be fully inhibited by the antioxidant Trolox (Keynes et al., 2005) with any residual consumption attributed to red blood cell contamination. We tested six of the most potent compounds (at 10 μM) for their ability to inhibit NO consumption in cerebellar cell suspensions. All the compounds tested substantially inhibited NO consumption in this model (Figure 1) such that the NO levels approached those attained in buffer, the residual “shoulder” seen after 1–2 min being due to red blood cells.

### 3.3 Comparison with established antioxidants

The link between cellular NO consumption and lipid peroxidation prompted direct studies of the antioxidant properties of the compounds. Earlier reports (Moosmann, Skutella, Beyer, & Behl, 2001; Yu et al., 1992) have investigated the structure–activity relationship of phenothiazine‐based compounds as antioxidants. In both investigations, it was reported that the absence of a substitution at N‐10 of the phenothiazine ring was required for potent activity. Methylation of the ring at N‐10 rendered the compound much less active. We tested the methylated and “native” phenothiazine compounds alongside a range of potent NO consumption inhibitors (compounds **13**,** 15**,** 26**,** 27**,** 53**, and **61**), as well as the antioxidant Trolox and a clinically approved free radical scavenger edaravone (Green & Ashwood, 2005). The mechanism of edaravone is thought to depend on the presence of the edaravone anion's ability to neutralize radicals (Higashi, Jitsuiki, Chayama, & Yoshizumi, 2006).

In addition to testing NO consumption by oxyhemoglobin assay, the degree of inhibition of lipid peroxidation in the preparation was measured in parallel using the TBARS assay (Esterbauer & Cheeseman, 1990). The unsubstituted phenothiazine and all novel compounds showed a very similar potency in both assays and were up to 1,000 times more potent than edaravone, Trolox, and the N‐methylated phenothiazine (Table 3).

**Table 3:** Comparison of compounds in NO consumption and TBARS assays

Compound	Structure	NO consumption IC_50_ (μM)	TBARS IC_50_ (μM)
Phenothiazine	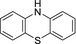	0.015 ± 0.001	0.042 ± 0.006
N‐Me‐phenothiazine	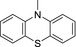	4.53 ± 3.4	18.6 ± 6.2
Edaravone	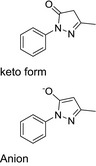	4.16 ± 2.1	29.2 ± 3.7
Trolox	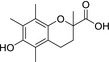	10.01 ± 5.8	19.2 ± 3.1
13	Structures in Table S1	0.10 ± 0.036	0.15 ± 0.002
15		0.084 ± 0.023	0.23 ± 0.01
26^a^		0.014 ± 0.013	0.10 ± 0.01
27^a^		0.099 ± 0.065	0.30 ± 0.058
53^a^		0.030 ± 0.004	0.038 ± 0.002
61^a^		0.023 ± 0.004	0.087 ± 0.006

Phenothiazine‐based compounds with an N‐10 substituent. All experiments carried out using the same drug solutions and membrane preparations.

### 3.4 DT‐PTZ‐C is a potent antioxidant

**Figure 2:**
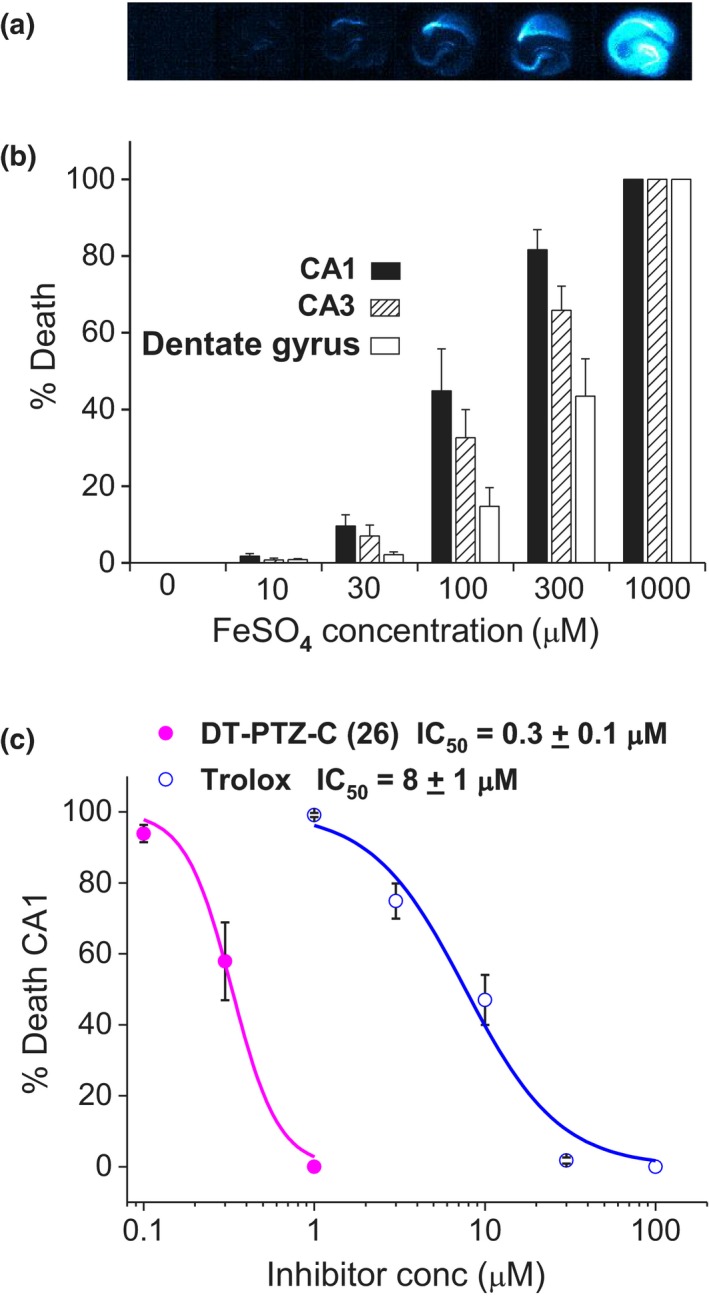
DT‐PTZ‐C inhibits iron/ascorbate toxicity with greater potency than Trolox. (a) Representative photomicrographs of PI‐stained hippocampal slices 24 hr following exposure to ascorbate (500 μM) and increasing concentrations of FeSO_4_ (0–1 mM). (b) Summary data (mean ± *SEM*;* n* = 8 slices) are expressed as percentage death in the three major hippocampal regions (CA1, CA3, and dentate gyrus). (c) The concentration‐dependent effects of DT‐PTZ‐C and Trolox were assessed against maximum slice toxicity (500 μM ascorbate + 1 mM iron) and are expressed as mean ± SEM % death in CA1, *n *=* *8 slices

Such potent inhibitors of NO consumption (and lipid peroxidation) should protect intact tissue against cell death induced by oxidative stress. Hippocampal slice cultures were subjected to incubation with ascorbate (500 μM) and FeSO_4_ (10–1,000 μM), and toxicity was assessed by PI staining after 24 hr. A similar model has been used before (Liu, Liu, Doctrow, & Baudry, 2003). As the FeSO_4_ concentration increased, the percentage of cell death in all areas of the slice increased, such that by 100 μM FeSO_4_, death in CA1 was ~50% (Fig 2a, b). As seen previously with this preparation (Keynes et al., 2004), neuronal death was graded in different slice regions, in the order of CA1 > CA3 > dentate gyrus. Staining was maximum (100% death) in all areas following treatment with 1 mM FeSO_4_.

DT‐PTZ‐C (0.1–1 μM) and the reference antioxidant Trolox (1–100 μM) were tested in slices undergoing treatment with ascorbate (500 μM) and FeSO_4_ (1 mM). Both compounds prevented slice toxicity across all regions in a concentration‐dependent manner. Measured as percentage death in CA1, IC_50_ values were 0.3 ± 0.1 μM for compound DT‐PTZ‐C and 8 ± 1 μM for Trolox (Figure 2c).

**Figure 3:**
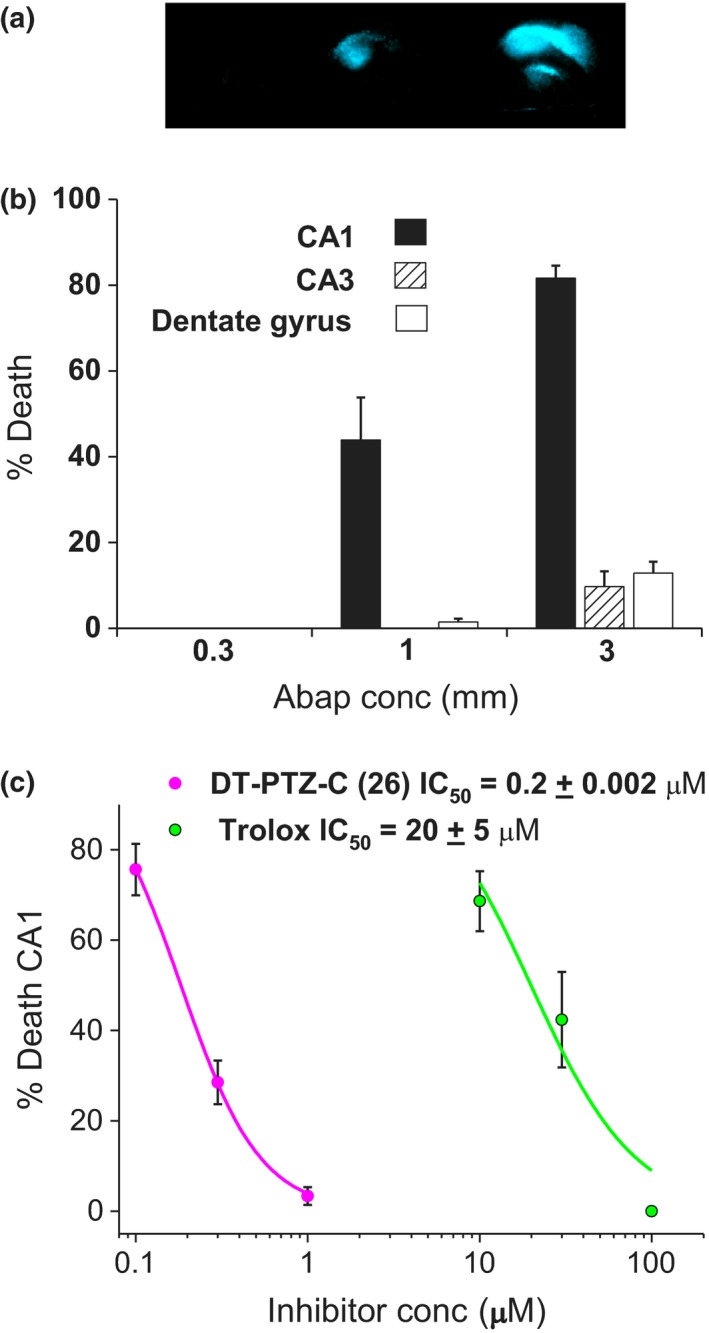
DT‐PTZ‐C inhibits ABAP toxicity with greater potency than Trolox. (a) Representative photomicrographs of hippocampal slices stained with PI 24 h following exposure to ABAP (0.3–3 mM). (b) Summary data (mean ± *SEM*;* n* = 8 slices) are expressed as percentage death in CA1. (c) The concentration‐dependent effects of DT‐PTZ‐C and Trolox were assessed against maximum slice toxicity (3 mM ABAP) and are expressed as mean ± *SEM* % death in CA1, *n *=* *8 slices

In a second model of lipid peroxidation‐induced neuronal death, the “Azo” initiator ABAP (0.3–3 mM) was administered to slice cultures for 24 hr and death measured as above. Treatment with this compound elicited slice toxicity more selectively in CA1. Following 1 mM ABAP treatment, CA1 death was 44 ± 10%, increasing to 82 ± 3% with 3 mM (Fig 3a, b). Both test compounds prevented slice toxicity following a challenge with 3 mM ABAP, with IC_50_ values of 0.2 ± 0.002 μM for DT‐PTZ‐C and 20 ± 5 μM for Trolox (Figure 3c).

### 3.5 Compound **26** inhibits NO consumption with similar potency to other aromatic imines

**Figure 4:**
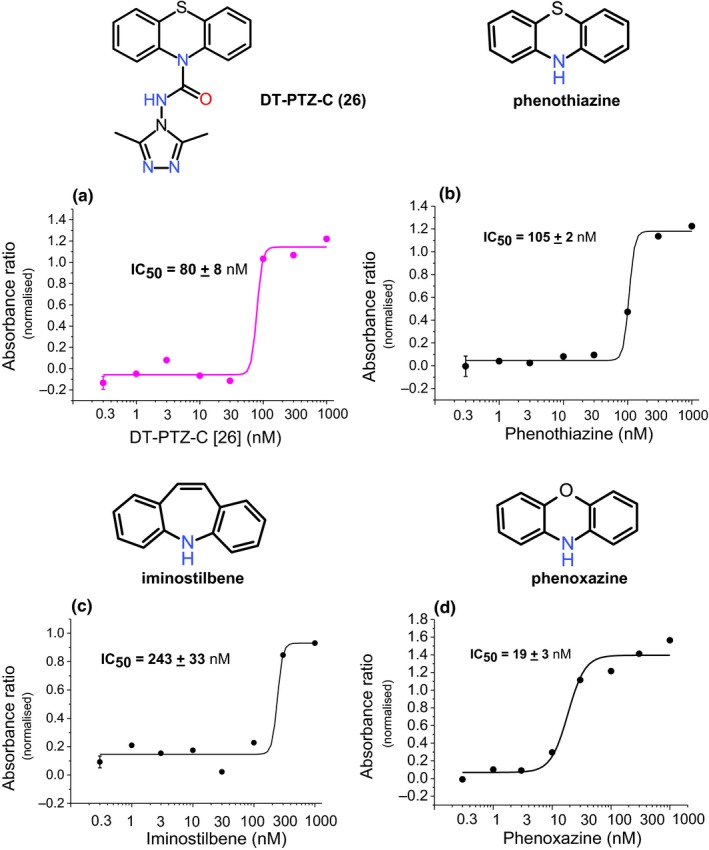
DT‐PTZ‐C inhibits NO consumption with a similar potency to aromatic imines. Summary data (mean ± *SEM*;* n* = 4) of hemoglobin‐coated bead absorbance following incubation for 25 min with pellet (0.1 mg/ml), supernatant (10%), and DETA/NO (100 μM). Increasing concentrations (0.3–1,000 nM) of (a) compound **26**, (b) phenothiazine, (c) iminostilbene, and (d) phenoxazine were included to determine their potency at inhibiting NO consumption

In common with the other potent inhibitors of NO consumption identified here, DT‐PTZ‐C is based upon a phenothiazine structure. A previous report found the aromatic imines phenothiazine, phenoxazine, and iminostilbene were highly potent antioxidant compounds in several neuroprotective models (Moosmann et al., 2001). The ability of these compounds to inhibit NO consumption was tested using the oxyhemoglobin bead assay and compared to compound **26**. Compounds inhibited NO consumption with the following IC_50_′s: compound **26**, 80 ± 8 nM; phenothiazine, 105 ± 2 nM; iminostilbene 243 ± 33 nM; and phenoxazine, 19 ± 3 nM (Figure 4a–d).

### 3.6 Structure–activity relationships and electrostatic fields

**Figure 5:**
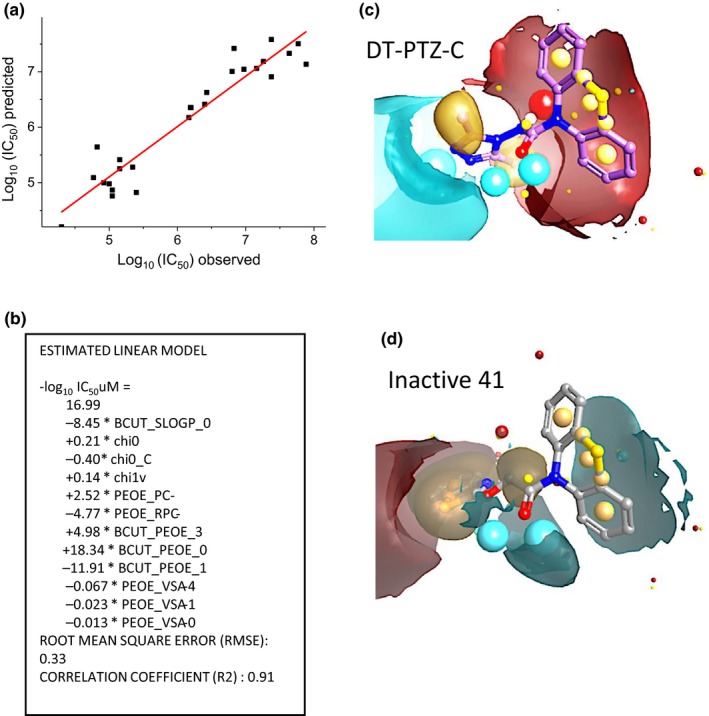
(a) QSAR model of antioxidant activity using calculated descriptors. (b). Details of the model. (c) DT‐PTZ‐C field analysis using Cresset. Negative field points—blue; positive field points—dark red; van der Waals surface field points—yellow, hydrophobic field points—gold/orange. (d) Field analysis of a weakly active compound **41**

In order to better understand the structural features important for activity, we conducted a QSAR analysis of the dataset. The analysis utilized all the available IC_50_ data (see tables), and multivariate partial least squares (PLS) analysis produced a good model with a R‐squared (*R* ^2^) of 0.907. The model (Figure 4 and 5panels A, B) showed a high dependence on electronegative descriptors notably PEOE (partial equalization of orbital electronegativity; Gasteiger & Marsili, 1980). The major descriptor, BCUT_PEOE_0, is considered a chemistry‐space descriptor where BCUT is calculated from an eigenvalue of an adjacency matrix and PEOE is a partial charge (Pearlman & Smith, 1998). Two other types of PEOE descriptors also contribute to the model (Table 4). One descriptor, BCUT_SLOGP_0, showed a lipophilicity contribution (0.62). Molecular shape was also a contributor as indicated by the chi descriptors. The importance of electronegativity was further highlighted by a molecular field analysis (Figure 5panels C, D) using the Cresset software XED force field. It is assumed that similar fields will belong to the molecules with similar properties (Cheeseright et al., 2006). Cresset represents the molecule, using the four molecular fields (Rose & Vinter, 2007): positive electrostatic, negative electrostatic, van der Waals attractive (“steric”), and hydrophobic. Comparison of the best active molecule DT‐PTZ‐C with a weak active 41 (panel D) shows a clear qualitative difference between the electrostatic fields (negative field points—blue; positive field points—dark red). This model is consistent with the ability of the molecules to generate a stable cation‐radical species following SET by a reactive radical (vide infra).

**Table 4:** Relative importance of descriptors and type

Descriptor	Brief description of properties
*0.62 BCUT_SLOGP_0*	BCUT descriptors using atomic contribution to logP (using the Wildman and Crippen SlogP method(Wildman & Crippen, 1999)) instead of partial charge
*0.25 chi0*	Chi connectivity indices (Hall & Kier, 2007) are calculated from the heavy atom degree di (number of heavy neighbors) and vi. These capture different aspects of molecular shape. Chi1v is an atomic valence connectivity index
*0.31 chi0_C*	
*0.079 chi1v*	
*0.37 PEOE_PC‐*	The descriptors implement the partial equalization of orbital electronegativities (PEOE) method of calculating atomic partial charges (Gasteiger & Marsili, 1980)
*0.20 PEOE_RPC‐*	
*0.27 BCUT_PEOE_3*	BCUT descriptors (Pearlman & Smith, 1998) are calculated from the eigenvalues of a modified adjacency matrix. The diagonal takes the value of the PEOE partial charges
*1.00 BCUT_PEOE_0*	
*0.18 BCUT_PEOE_1*	
*0.25 PEOE_VSA‐4*	Descriptors of fractional polar van der Waals surface area
*0.16 PEOE_VSA‐1*	
*0.14 PEOE_VSA‐0*	

A brief description of the descriptors is given. More detail is available in the MOE application notes.

### 3.7 The oxyhemoglobin bead assay is a simple test for antioxidant compounds

**Figure 6:**
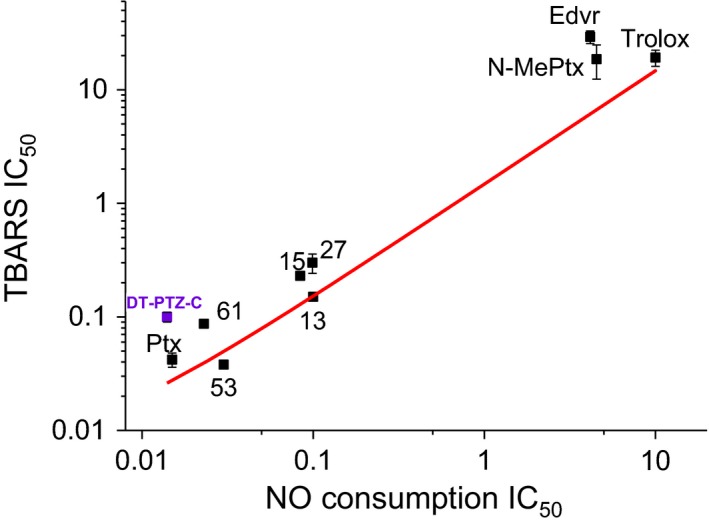
Comparison of IC_50_'s for phenothiazine‐based compounds in the hemoglobin bead versus TBARS assays. (a) A significant correlation between the two assays was found (Pearson's rank correlation coefficient, *r* = 0.92)

Using a variation of the well‐known oxyhemoglobin assay (Livingston, 1996), compounds were systematically screened for antioxidant properties by measuring inhibition of NO consumption in the presence of brain fractions undergoing peroxidation. In agreement with results determined by other methods (Jeding et al., 1995; Moosmann et al., 2001; Yu et al., 1992), phenothiazine‐based compounds were found to have antioxidant properties. To evaluate this novel use of a hemoglobin‐based NO detection assay, IC_50_'s for a number of compounds were compared with those determined using the TBARS assay (Figure 6).

It is evident from this comparison that inhibition of NO consumption detected by the oxyhemoglobin bead assay is highly predictive of antioxidant potency for the range of phenothiazine compounds tested. The bead assay represents a simple, cheap, and reproducible method of determining antioxidant properties, and one which does not require the use of hazardous chemicals.

## 4 DISCUSSION

### 4.1 NO consumption inhibitors are potent antioxidants

We tested six of the NO consumption inhibitors in an intact cellular model of lipid peroxidation (cerebellar cells, see Figure 1) and found all to approach 100% inhibition at 10 μM. In this model, remaining NO consumption is likely due to red blood cell contamination (Keynes et al., 2005). The possibility that these compounds may protect intact brain tissue from cell death induced by oxidative stress was explored using hippocampal slice cultures. Of the most potent inhibitors identified, DT‐PTZ‐C was chosen for testing as it has no effects on the NO‐producing enzyme (nNOS) or on the activity of the guanylyl cyclase‐linked NO receptor (also known as soluble guanylyl cyclase) and has only a slight tendency to be pro‐oxidant (oxidizing hemoglobin beads with an EC_50_ of 1.1 μM) compared to its low nM potency at inhibiting NO consumption (data not shown). Under both test conditions (iron/ascorbate or “Azo” initiated slice toxicity), DT‐PTZ‐C was extremely potent (IC_50_ <300 nM) compared to the Trolox control, and both compounds afforded complete protection of the slices. In comparison, a similar study reported only a 50–70% increase in slice survival with 20 μM of the SOD/catalase mimetic EUK‐134 (Liu et al., 2003), though no control compound was included for comparison.

### 4.2 Novel use for an old structure

Since the antioxidant potential of the phenothiazine‐based neuroleptic drugs chlorpromazine, promethazine, and methotrimeprazine (Table 2) has been reported before (Jeding et al., 1995), the ability of other phenothiazines to prevent lipid peroxidation is perhaps not surprising. Jeding et al. investigated the mechanism by which such phenothiazines act, finding that while none of the drugs reacted with O_2_^**•−**^, chlorpromazine and methotrimeprazine were powerful (almost diffusion controlled) •OH scavengers, and all three compounds were powerful inhibitors of iron‐dependent lipid peroxidation and peroxyl radical scavengers. In addition, chlorpromazine showed some ability to bind iron ions. During the review process, a referee highlighted the difference between the experimental systems (where the ability of antioxidants to intercept •OH is well known) and the in vivo situation where this may not be the case. Several studies report, for example, the phenothiazine blockade of •OH as produced by 6‐aminodopamine in a xanthine oxidase xanthine experimental system (Heikkila, Cohen, & Manian, 1975) and also with the Fenton reaction (Borges et al., 2010). Phenothiazines are lipophilic and partition preferentially into membranes; as electron transfer can occur over some distance, interception of •OH may not be impossible (Kuss‐Petermann & Wenger, 2016). Of course, administered drugs do not approach the concentration of bulk membrane and the formation of lipid peroxyl radicals from •OH may be the dominant pathway in vivo.

An earlier study had investigated the structural basis upon which phenothiazine‐centered compounds may act as iron‐dependent lipid peroxidation inhibitors (Yu et al., 1992). The authors varied many structural components, concluding that the most important determinant of potent in vitro lipid peroxidation inhibitory activity was the absence of a substitution at N‐10 of the phenothiazine ring, since its methylation rendered the compound markedly less active. In addition, they found that the parent phenothiazine nucleus exhibited good inhibitory action.

A more recent study by Moosmann et al. (2001) investigated different aromatic amine and imine compounds for their neuroprotective action in several cell culture paradigms including clonal cell lines, primary cerebellar neurons and hippocampal slice cultures. They noted that antioxidant properties are increased when the two benzene rings are bridged with a sulfur or oxygen. Similarly to the study of Yu et al. (1992), the authors found N‐methyl‐phenothiazine to be significantly less potent compared with phenothiazine alone. They concluded that the presence of at least one single NH‐bond is an essential pre‐requisite for antioxidant action, and the compound's activity is likely to stem from the dissociation of this bond, leading to an imine radical. Moosmann et al. reported that three structurally similar compounds phenothiazine, phenoxazine, and iminostilbene had IC_50_'s of 20–75 nM in their experimental paradigms. Separate studies on the radical trapping activity of unsubstituted N‐10 phenoxazines demonstrated an ability to trap more two or more peroxyl radicals indicating the potential for this group of compounds (Farmer, Haidasz, Griesser, & Pratt, 2017; Lucarini et al., 1999).

In agreement with the work of Moosmann, we also found low nM potency for the effect of phenothiazine, phenoxazine, and iminostilbene as inhibitors of NO consumption/lipid peroxidation (Figure 4). Again in agreement with both Moosmann and Yu, we found the N‐methylated phenothiazine was significantly less potent than phenothiazine alone (Table 3). The observation that compounds **26**,** 27**,** 40**,** 53**, and **61** (and others in the series), all of which have N^10^‐carbamoyl‐substituents (see Table 3), are highly potent was, however, entirely unpredictable from the present literature.

**Figure 7:**
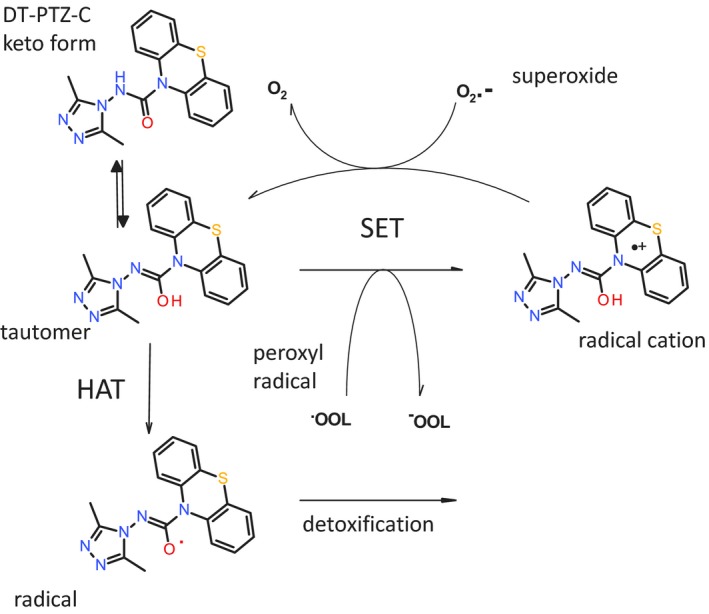
Lipid peroxidation activity of phenothiazines proceeds via generation of a radical cation or potentially a radical (drawn as oxygen centered). Stabilization of the structures is proposed to occur via the tautomeric form

In considering possible mechanisms of the antioxidant effect, the ability of phenothiazines to donate electrons seems to be key. Likely mechanisms include both single‐electron transfer (SET) and hydrogen atom transfer (HAT) possibilities (Figure 7).

The radical cation of phenothiazines is easily the lowest energy intermediate when compared with the phenothiazine radical. In addition, phenothiazines are not thought to be good hydrogen atom donors (Zhu, Dai, Yu, Wu, & Cheng, 2008). The phenothiazine radical is little reported in the literature, and when formed chemically, it degrades to produce highly colored degradation products (Hanson & Norman, 1973). Our favored mechanism requires that the radical cation be generated in lipids. Phenothiazine radical cations have been observed in micelles (Nemcova, Novotny, & Horska, 1986) and in model membranes and mitochondria (Borges et al., 2010). The theoretical assumption is that a charged cation species is being generated in a non‐polar environment (the lipid); however in reality, membranes contain small polar molecules including water as well as other species such as cholesterol and proteins. If we draw the structure of DT‐PTZ‐C as the tautomer (Figure 7), then it is apparent that further stabilization of the radical cation or the radical form is possible. In the case of molecules such as phenols and enolates, the most facile reaction available is the HAT (Jovanovic, Steenken, Boone, & Simic, 1999) and has also been invoked to explain the antioxidant activity of phenoxazines (Farmer et al., 2017). It is clear that defining the mechanism of action of DT‐PTZ‐C will require further research, which is beyond the scope of this study.

How does the N^10^ carbamoyl group stabilize the proposed radical or radical cation intermediate? Hammett values for electron withdrawing ability of carbamoyl indicate a σ_*F*_ value of 0.23 and σ_*R*_ of 0.12; this compares with 0.54 and 0.12, respectively, for the electronegative group CN (Hansch, Leo, & Taft, 1991). In addition, we should consider that the best compound DT‐PTZ‐C is in fact hydrazide like and will therefore be even less electron withdrawing. Drawing the molecule in its tautomeric form (Figure 7) highlights how extended conjugation could influence stability of the radical cation.

Finally, the structure–activity relationships could be quantitatively explained by a model that included chemical descriptors of electronegative properties.

### 4.3 N‐carbamoylphenothiazines may be superior antioxidants

Oxidative stress has been widely linked to many disease states, where ferroptosis regulated cell death is implicated. In particular chronic neurodegeneration such as Alzheimer's disease (Behl & Moosmann, 2002), Parkinson's disease, amyotrophic lateral sclerosis (ALS; Pollari, Goldsteins, Bart, Koistinaho, & Giniatullin, 2014), and multiple sclerosis (Gilgun‐Sherki, Melamed, & Offen, 2004), or more acute insults such as ischemia (Green & Ashwood, 2005). While there is much evidence for the efficacy of direct‐acting antioxidants in animal models of these diseases, the clinical evidence that antioxidant compounds can be neuroprotective has been relatively scarce (Gilgun‐Sherki et al., 2004; Moosmann & Behl, 2002). However, edaravone has recently been approved for acute ischemic stroke in Japan (Miyaji et al., 2015) and for the treatment of ALS, albeit in a well‐defined subset of patients (Abe et al., 2017). Edaravone must be delivered by intravenous infusion, and blood–brain barrier penetration may not be ideal (Fang et al., 2014). In our hands, edaravone is a relatively weak antioxidant (Table 3). N^10^‐alkylphenothiazine drugs such as chlorpromazine and promethazine have been advanced as potential stroke therapies (S. M. Liu et al., 2015). Clearly, there is considerable scope for improved compounds to advance to the clinic.

The N‐substituted phenothiazine antioxidant compounds outlined here are 100‐fold more potent as antioxidants than simple N^10^‐alkylphenothiazine drugs or edaravone, they are of low molecular weight (an advantage for blood–brain barrier permeability) and have other drug‐like chemical characteristics. We identified DT‐PTZ‐C as having high potency both in simple antioxidant systems and in hippocampal slice. This molecule also showed good selectivity with no observable activity against NO synthase or the guanylyl cyclase‐linked NO receptor and minimal pro‐oxidant properties. Optimized compounds may prove of value in the treatment of neurodegenerative conditions and also of the host of conditions involving oxidative stress outside the central nervous system (Cobley et al., 2018).

## Data Availability

Data available on request from the authors. Data are available in the supplementary information.

## References

[cbdd13572-bib-0001] Abe, K. , Aoki, M. , Tsuji, S. , Itoyama, Y. , Sobue, G. , Togo, M. , … Grp, E. M.‐A. S. (2017). Safety and efficacy of edaravone in well defined patients with amyotrophic lateral sclerosis: A randomised, double‐blind, placebo‐controlled trial. Lancet Neurology, 16(7), 505–512. 10.1016/S1474-4422(17)30115-1 28522181

[cbdd13572-bib-0002] Behl, C. , & Moosmann, B. (2002). Antioxidant neuroprotection in Alzheimer's disease as preventive and therapeutic approach. Free Radical Biology and Medicine, 33(2), 182–191. 10.1016/S0891-5849(02)00883-3 12106814

[cbdd13572-bib-0003] Borges, M. B. D. , Dos Santos, C. G. , Yokomizo, C. H. , Sood, R. , Vitovic, P. , Kinnunen, P. K. J. , … Nantes, I. L. (2010). Characterization of hydrophobic interaction and antioxidant properties of the phenothiazine nucleus in mitochondrial and model membranes. Free Radical Research, 44(9), 1054–1063. 10.3109/10715762.2010.498826 20815768

[cbdd13572-bib-0004] Cacabelos, D. , Ayala, V. , Ramirez‐Nunez, O. , Granado‐Serrano, A. B. , Boada, J. , Serrano, J. C. , … Portero‐Otin, M. (2014). Dietary lipid unsaturation influences survival and oxidative modifications of an amyotrophic lateral sclerosis model in a gender‐specific manner. Neuromolecular Medicine, 16(4), 669–685. 10.1007/s12017-014-8317-7 24980941

[cbdd13572-bib-0005] Callaway, J. K. , Beart, P. M. , & Jarrott, B. (1998). A reliable procedure for comparison of antioxidants in rat brain homogenates. Journal of Pharmacological and Toxicological Methods, 39(3), 155–162. 10.1016/S1056-8719(98)00022-7 9741390

[cbdd13572-bib-0006] Cheeseright, T. , Mackey, M. , Rose, S. , & Vinter, A. (2006). Molecular field extrema as descriptors of biological activity: Definition and validation. Journal of Chemical Information and Modeling, 46(2), 665–676. 10.1021/ci050357s 16562997

[cbdd13572-bib-0008] Cobley, J. N. , Fiorello, M. L. , & Bailey, D. M. (2018). 13 reasons why the brain is susceptible to oxidative stress. Redox Biology, 15, 490–503. 10.1016/j.redox.2018.01.008 29413961 PMC5881419

[cbdd13572-bib-0009] Dixon, S. J. , Lemberg, K. M. , Lamprecht, M. R. , Skouta, R. , Zaitsev, E. M. , Gleason, C. E. , … Stockwell, B. R. (2012). Ferroptosis: An iron‐dependent form of nonapoptotic cell death. Cell, 149(5), 1060–1072. 10.1016/j.cell.2012.03.042 22632970 PMC3367386

[cbdd13572-bib-0010] Durant, J. L. , Leland, B. A. , Henry, D. R. , & Nourse, J. G. (2002). Reoptimization of MDL keys for use in drug discovery. Journal of Chemical Information and Computer Sciences, 42(6), 1273–1280. 10.1021/ci010132r 12444722

[cbdd13572-bib-0011] Esterbauer, H. , & Cheeseman, K. H. (1990). Determination of aldehydic lipid‐peroxidation products ‐ malonaldehyde and 4‐hydroxynonenal. Methods in Enzymology, 186, 407–421. 2233308 10.1016/0076-6879(90)86134-h

[cbdd13572-bib-0012] Fang, W. R. , Zhang, R. , Sha, L. , Lv, P. , Shang, E. X. , Han, D. , … Li, Y. M. (2014). Platelet activating factor induces transient blood‐brain barrier opening to facilitate edaravone penetration into the brain. Journal of Neurochemistry, 128(5), 662–671. 10.1111/jnc.12507 24164378

[cbdd13572-bib-0013] Farmer, L. A. , Haidasz, E. A. , Griesser, M. , & Pratt, D. A. (2017). Phenoxazine: A privileged scaffold for radical‐trapping antioxidants. Journal of Organic Chemistry, 82(19), 10523–10536. 10.1021/acs.joc.7b02025 28885854

[cbdd13572-bib-0014] Forman, H. J. , Augusto, O. , Brigelius‐Flohe, R. , Dennery, P. A. , Kalyanaraman, B. , Ischiropoulos, H. , … Davies, K. J. A. (2015). Even free radicals should follow some rules: A Guide to free radical research terminology and methodology. Free Radical Biology and Medicine, 78, 233–235. 10.1016/j.freeradbiomed.2014.10.504 25462642

[cbdd13572-bib-0015] Friedmann Angeli, J. P. , Schneider, M. , Proneth, B. , Tyurina, Y. Y. , Tyurin, V. A. , Hammond, V. J. , … Conrad, M. (2014). Inactivation of the ferroptosis regulator Gpx4 triggers acute renal failure in mice. Nature Cell Biology, 16(12), 1180–1191. 10.1038/ncb3064 25402683 PMC4894846

[cbdd13572-bib-0016] Galluzzi, L. , Vitale, I. , Aaronson, S. A. , Abrams, J. M. , Adam, D. , Agostinis, P. , … Kroemer, G. (2018). Molecular mechanisms of cell death: Recommendations of the Nomenclature Committee on Cell Death 2018. Cell Death and Differentiation, 25(3), 486–541. 10.1038/s41418-017-0012-4 29362479 PMC5864239

[cbdd13572-bib-0017] Garthwaite, J. , & Garthwaite, G. (1987). Cellular‐origins of cyclic‐Gmp responses to excitatory amino‐acid receptor agonists in rat cerebellum in vitro. Journal of Neurochemistry, 48(1), 29–39. 10.1111/j.1471-4159.1987.tb13123.x 2878975

[cbdd13572-bib-0018] Gaschler, M. M. , & Stockwell, B. R. (2017). Lipid peroxidation in cell death. Biochemical and Biophysical Research Communications, 482(3), 419–425. 10.1016/j.bbrc.2016.10.086 28212725 PMC5319403

[cbdd13572-bib-0019] Gasteiger, J. , & Marsili, M. (1980). Iterative partial equalization of orbital electronegativity ‐ a rapid access to atomic charges. Tetrahedron, 36(22), 3219–3228. 10.1016/0040-4020(80)80168-2

[cbdd13572-bib-0020] Gilgun‐Sherki, Y. , Melamed, E. , & Offen, D. (2004). The role of oxidative stress in the pathogenesis of multiple sclerosis: The need for effective antioxidant therapy. Journal of Neurology, 251(3), 261–268. 10.1007/s00415-004-0348-9 15015004

[cbdd13572-bib-0021] Green, A. R. , & Ashwood, T. (2005). Free radical trapping as a therapeutic approach to neuroprotection in stroke: Experimental and clinical studies with NXY‐059 and free radical scavengers. Current Drug Targets: CNS & Neurological Disorders, 4(2), 109–118. 15857295 10.2174/1568007053544156

[cbdd13572-bib-0022] Hall, C. N. , & Garthwaite, J. (2006). Inactivation of nitric oxide by rat cerebellar slices. Journal of Physiology, 577(Pt 2), 549–567. 10.1113/jphysiol.2006.118380 16973697 PMC1890435

[cbdd13572-bib-0023] Hall, L. H. K. , & Kier, L. B. (2007). The Molecular Connectivity Chi Indexes and Kappa Shape Indexes in Structure‐Property Modeling In Lipkowitz K. B. B., & Boyd D. B. (Eds.), Reviews in Computational Chemistry. Hoboken, NJ: Wiley.

[cbdd13572-bib-0024] Hambright, W. S. , Fonseca, R. S. , Chen, L. , Na, R. , & Ran, Q. (2017). Ablation of ferroptosis regulator glutathione peroxidase 4 in forebrain neurons promotes cognitive impairment and neurodegeneration. Redox Biology, 12, 8–17. 10.1016/j.redox.2017.01.021 28212525 PMC5312549

[cbdd13572-bib-0025] Hansch, C. , Leo, A. , & Taft, R. W. (1991). A survey of Hammett substituent constants and resonance and field parameters. Chemical Reviews, 91(2), 165–195. 10.1021/cr00002a004

[cbdd13572-bib-0026] Hanson, P. , & Norman, R. O. C. (1973). Heterocyclic free‐radicals. 4. Some reactions of phenothiazine, 2 derived radicals, and phenothiazin‐5‐ium ion. Journal of the Chemical Society‐Perkin Transactions, 2(3), 264–271. 10.1039/p29730000264

[cbdd13572-bib-0027] Heikkila, R. E. , Cohen, G. , & Manian, A. A. (1975). Reactivity of various phenothiazine derivatives with oxygen and oxygen radicals. Biochemical Pharmacology, 24(3), 363–368. 10.1016/0006-2952(75)90219-1 1125042

[cbdd13572-bib-0028] Higashi, Y. , Jitsuiki, D. , Chayama, K. , & Yoshizumi, M. (2006). Edaravone (3‐methyl‐1‐phenyl‐2‐pyrazolin‐5‐one), a novel free radical scavenger, for treatment of cardiovascular diseases. Recent Advances in Cardiovascular Drug Discovery, 1(1), 85–93. 10.2174/157489006775244191 18221078

[cbdd13572-bib-0029] Hu, C. L. , Nydes, M. , Shanley, K. L. , Morales Pantoja, I. E. , Howard, T. A. , & Bizzozero, O. A. (2018). Reduced expression of the ferroptosis inhibitor glutathione peroxidase‐4 in multiple sclerosis and experimental autoimmune encephalomyelitis. Journal of Neurochemistry, 148(3), 426–439. 10.1111/jnc.14604 30289974 PMC6347488

[cbdd13572-bib-0030] Jeding, I. , Evans, P. J. , Akanmu, D. , Dexter, D. , Spencer, J. D. , Aruoma, O. I. , … Halliwell, B. (1995). Characterization of the potential antioxidant and prooxidant actions of some neuroleptic drugs. Biochemical Pharmacology, 49(3), 359–365. 10.1016/0006-2952(94)00424-K 7857323

[cbdd13572-bib-0031] Jovanovic, S. V. , Steenken, S. , Boone, C. W. , & Simic, M. G. (1999). H‐atom transfer is a preferred antioxidant mechanism of curcumin. Journal of the American Chemical Society, 121(41), 9677–9681. 10.1021/ja991446m

[cbdd13572-bib-0032] Keynes, R. G. , Duport, S. , & Garthwaite, J. (2004). Hippocampal neurons in organotypic slice culture are highly resistant to damage by endogenous and exogenous nitric oxide. European Journal of Neuroscience, 19(5), 1163–1173. 10.1111/j.1460-9568.2004.03217.x 15016075

[cbdd13572-bib-0033] Keynes, R. G. , Griffiths, C. H. , Hall, C. , & Garthwaite, J. (2005). Nitric oxide consumption through lipid peroxidation in brain cell suspensions and homogenates. Biochemical Journal, 387, 685–694. 10.1042/Bj20041431 15579136 PMC1134998

[cbdd13572-bib-0034] Koppenol, W. H. (2001). The Haber‐Weiss cycle ‐ 70 years later. Redox Report, 6(4), 229–234. 10.1179/135100001101536373 11642713

[cbdd13572-bib-0035] Kuss‐Petermann, M. , & Wenger, O. S. (2016). Unusual distance dependences of electron transfer rates. Physical Chemistry Chemical Physics, 18(28), 18657–18664. 10.1039/c6cp03124b 27353891

[cbdd13572-bib-0036] Lewerenz, J. , Ates, G. , Methner, A. , Conrad, M. , & Maher, P. (2018). Oxytosis/ferroptosis‐(Re‐) emerging roles for oxidative stress‐dependent non‐apoptotic cell death in diseases of the central nervous system. Frontiers in Neuroscience, 12, 214 10.3389/fnins.2018.00214 29731704 PMC5920049

[cbdd13572-bib-0037] Liu, S. M. , Geng, X. K. , Forreider, B. , Xiao, Y. , Kong, Q. T. , Ding, Y. C. , & Ji, X. M. (2015). Enhanced beneficial effects of mild hypothermia by phenothiazine drugs in stroke therapy. Neurological Research, 37(5), 454–460. 10.1179/1743132815y.0000000031 25819773

[cbdd13572-bib-0038] Liu, R. L. , Liu, W. , Doctrow, S. R. , & Baudry, M. (2003). Iron toxicity in organotypic cultures of hippocampal slices: Role of reactive oxygen species. Journal of Neurochemistry, 85(2), 492–502. 10.1046/j.1471-4159.2003.01708.x 12675926

[cbdd13572-bib-0039] Livingston, K. (1996). Methods in nitric oxide research ‐ Feelisch, M, Stamler, JS. Science, 272(5265), 1117.

[cbdd13572-bib-0040] Lucarini, M. , Pedrielli, P. , Pedulli, G. F. , Valgimigli, L. , Gigmes, D. , & Tordo, P. (1999). Bond dissociation energies of the N‐H bond and rate constants for the reaction with alkyl, alkoxyl, and peroxyl radicals of phenothiazines and related compounds. Journal of the American Chemical Society, 121(49), 11546–11553. 10.1021/ja992904u

[cbdd13572-bib-0041] Mattson, M. P. (1998). Modification of ion homeostasis by lipid peroxidation: Roles in neuronal degeneration and adaptive plasticity. Trends in Neurosciences, 21(2), 53–57. 10.1016/S0166-2236(97)01188-0 9498297

[cbdd13572-bib-0042] Miyaji, Y. , Yoshimura, S. , Sakai, N. , Yamagami, H. , Egashira, Y. , Shirakawa, M. , … Tomogane, Y. (2015). Effect of Edaravone on favorable outcome in patients with acute cerebral large vessel occlusion: Subanalysis of RESCUE‐Japan Registry. Neurologia Medico‐Chirurgica, 55(3), 241–247. 10.2176/nmc.ra.2014-0219 25739433 PMC4533339

[cbdd13572-bib-0043] Moosmann, B. , & Behl, C. (2002). Antioxidants as treatment for neurodegenerative disorders. Expert Opinion on Investigational Drugs, 11(10), 1407–1435. 10.1517/13543784.11.10.1407 12387703

[cbdd13572-bib-0044] Moosmann, B. , Skutella, T. , Beyer, K. , & Behl, C. (2001). Protective activity of aromatic amines and imines against oxidative nerve cell death. Biological Chemistry, 382(11), 1601–1612. 10.1515/Bc.2001.195 11767950

[cbdd13572-bib-0045] Nemcova, I. , Novotny, J. , & Horska, V. (1986). Spectrophotometric study of phenothiazine‐derivatives and their cation radicals in micellar media. Microchemical Journal, 34(2), 180–189. 10.1016/0026-265x(86)90030-5

[cbdd13572-bib-0046] Ohlow, M. J. , Sohre, S. , Granold, M. , Schreckenberger, M. , & Moosmann, B. (2017). Why have clinical trials of antioxidants to prevent neurodegeneration failed? ‐ A cellular investigation of novel phenothiazine‐type antioxidants reveals competing objectives for pharmaceutical neuroprotection. Pharmaceutical Research, 34(2), 378–393. 10.1007/s11095-016-2068-0 27896592

[cbdd13572-bib-0047] Ozbayer, C. , Kurt, H. , Nur Kebapci, M. , Veysi Gunes, H. , Colak, E. , & Degirmenci, I. (2018). The genetic variants of solute carrier family 11 member 2 gene and risk of developing type‐2 diabetes. Journal of Genetics, 97(5), 1407–1412. 30555088

[cbdd13572-bib-0048] Pearlman, R. S. , & Smith, K. M. (1998). Novel software tools for chemical diversity. Perspectives in Drug Discovery and Design, 9–11, 339–353. 10.1023/A:1027232610247

[cbdd13572-bib-0049] Pollari, E. , Goldsteins, G. , Bart, G. , Koistinaho, J. , & Giniatullin, R. (2014). The role of oxidative stress in degeneration of the neuromuscular junction in amyotrophic lateral sclerosis. Frontiers in Cellular Neuroscience, 8, 10.3389/fnce1.2014.00131 PMC4026683 24860432

[cbdd13572-bib-0050] Rose, S. , & Vinter, A. (2007). Molecular field technology and its applications in drug discovery. Innovations in Pharmaceutical Technology, 23, 14–18.

[cbdd13572-bib-0051] Steinhubl, S. R. (2008). Why have antioxidants failed in clinical trials? American Journal of Cardiology, 101(10a), 14d–19d. 10.1016/j.amjcard.2008.02.003 18474268

[cbdd13572-bib-0052] Stoppini, L. , Buchs, P. A. , & Muller, D. (1991). A simple method for organotypic cultures of nervous‐tissue. Journal of Neuroscience Methods, 37(2), 173–182. 10.1016/0165-0270(91)90128-M 1715499

[cbdd13572-bib-0053] Tan, S. , Schubert, D. , & Maher, P. (2001). Oxytosis: A novel form of programmed cell death. Current Topics in Medicinal Chemistry, 1(6), 497–506. 11895126 10.2174/1568026013394741

[cbdd13572-bib-0054] Tanimoto, T. T. (1958). An elementary mathematical theory of classification and prediction. New York, NY: International Business Machines Corp.

[cbdd13572-bib-0055] Tedesco, G. A 3D‐QSAR study on DPP‐4 inhibitors. https://www.cresset-group.com

[cbdd13572-bib-0056] Westerlund, C. , OstlundLindqvist, A. M. , Sainsbury, M. , Shertzer, H. G. , & Sjoquist, P. O. (1996). Characterization of novel indenoindoles. 1. Structure‐activity relationships in different model systems of lipid peroxidation. Biochemical Pharmacology, 51(10), 1397–1402. 10.1016/0006-2952(96)00080-9 8787557

[cbdd13572-bib-0057] Wildman, S. A. , & Crippen, G. M. (1999). Prediction of physicochemical parameters by atomic contributions. Journal of Chemical Information and Computer Sciences, 39(5), 868–873. 10.1021/ci990307l

[cbdd13572-bib-0058] Willett, P. , Barnard, J. M. , & Downs, G. M. (1998). Chemical similarity searching. Journal of Chemical Information and Computer Sciences, 38(6), 983–996. 10.1021/ci9800211

[cbdd13572-bib-0059] Yu, M. J. , Mccowan, J. R. , Thrasher, K. J. , Keith, P. T. , Luttman, C. A. , Ho, P. P. K. , … Saunders, R. D. (1992). Phenothiazines as lipid‐peroxidation inhibitors and cytoprotective agents. Journal of Medicinal Chemistry, 35(4), 716–724. 10.1021/jm00082a012 1542098

[cbdd13572-bib-0060] Zhu, X. Q. , Dai, Z. , Yu, A. , Wu, S. A. , & Cheng, J. P. (2008). Driving forces for the mutual conversions between phenothiazines and their various reaction intermediates in acetonitrile. Journal of Physical Chemistry B, 112(37), 11694–11707. 10.1021/jp8041268 18729403

