# Supplementary material for: N10‐carbonyl‐substituted phenothiazines inhibiting lipid peroxidation and associated nitric oxide consumption powerfully protect brain tissue against oxidative stress
Source: Chem Biol Drug Des. 2019 Jun 12;94(3):1680–93. doi: 10.1111/cbdd.13572 (PMC6790564; doi:10.1111/cbdd.13572)
Supplement: Supplementary file 1 [file CBDD-94-1680-s001.zip › cbdd13572-sup-0001-TableS1.docx]

**Supplementary information**

**N^10^-carbonyl-substituted phenothiazines inhibiting lipid peroxidation and associated nitric oxide consumption powerfully protect brain tissue against oxidative stress**

Robert G. Keynes, Anastasia Karchevskaya, Dieter Riddall, Charmaine H. Griffiths^,^ A.W. Edith Chan, David L. Selwood, and John Garthwaite.

**Contents**

1. **Commercial suppliers of DT-PTZ-C. Page 2**
2. **Supplementary table 1. Page 3-12**

**Commercial suppliers of** N-(3,5-dimethyl-4H-1,2,4-triazol-4-yl)-10H-phenothiazine-10-carboxamide, (DT-PTZ-C)

**Source Pubchem compound.**

[Mcule](https://pubchem.ncbi.nlm.nih.gov/source/Mcule)

PubChem SID: [165805597](https://pubchem.ncbi.nlm.nih.gov/substance/165805597)

Purchasable Chemical: [MCULE-7284203498](https://mcule.com/MCULE-7284203498/)

[TimTec](https://pubchem.ncbi.nlm.nih.gov/source/TimTec)

PubChem SID: [143656492](https://pubchem.ncbi.nlm.nih.gov/substance/143656492)

Purchasable Chemical: ST50104604

[Aurora Fine Chemicals LLC](https://pubchem.ncbi.nlm.nih.gov/source/Aurora%20Fine%20Chemicals%20LLC)

PubChem SID: [289713562](https://pubchem.ncbi.nlm.nih.gov/substance/289713562)

Purchasable Chemical: [K00.426.179](http://online.aurorafinechemicals.com/info?ID=K00.426.179)

[ZINC](https://pubchem.ncbi.nlm.nih.gov/source/ZINC)

PubChem SID: [257363698](https://pubchem.ncbi.nlm.nih.gov/substance/257363698)

Purchasable Chemical: [ZINC4671949](http://zinc.docking.org/substances/ZINC4671949/)

[Vitas-M Laboratory](https://pubchem.ncbi.nlm.nih.gov/source/Vitas-M%20Laboratory)

PubChem SID: [57730598](https://pubchem.ncbi.nlm.nih.gov/substance/57730598)

Purchasable Chemical: [STK081823](http://www.request.vitasmlab.biz/index.php?option=com_search_stk&Itemid=22&stk=STK081823&?utm_source=pubchem&utm_medium=p_search_link&utm_campaign=pubchem_search&utm_content=pubchem_slink)

[ChemDiv](https://pubchem.ncbi.nlm.nih.gov/source/ChemDiv)

PubChem SID: [329450875](https://pubchem.ncbi.nlm.nih.gov/substance/329450875)

Purchasable Chemical: [Y020-5222](http://chemistryondemand.com:8080/eShop/search_results.jsp?s_type=txt&idnumber=Y020-5222)

[Innovapharm](https://pubchem.ncbi.nlm.nih.gov/source/Innovapharm)

PubChem SID: [381653086](https://pubchem.ncbi.nlm.nih.gov/substance/381653086)

Purchasable Chemical: STT-00112509

**Supplementary Table 1.** Final round of screening of N-carbonyl substituted phenothiazines for NO consumption.

| **ID** | **Structural type** | **R1** | **R2** | **Molecular weight** | **ClogP** | **% Signal** | **Test**  **concentration** | **IC_50_** |
| --- | --- | --- | --- | --- | --- | --- | --- | --- |
| **1** | N^10^-carbonyl |  | H | 428.3 | 8.1 | 1 | 0.4 | 0.372 |
| **2** | N^10^-carbonyl |  | H | 497.6 | 3.0 | 98 | 0.4 |  |
| **3** | Chlorinated phenothiazine |   Full structure | - | 337.0 | 7.2 | 100 | 0.4 |  |
| **4** | Fused ring phenothiazine |   Full structure | - | 277.4 | 5.5 | 95 | 0.4 |  |
| **5** | N^10^-carbonyl |  | H | 339.4 | 3.7 | 95 | 0.4 |  |
| **6** | N^10^-carbonyl |  | H | 433.5 | 4.5 | 100 | 0.4 |  |
| **7** | N^10^-carbonyl |  | H | 362.4 | 3.16 | 0 | 0.4 | 0.148 |
| **9** | N^10^-carbonyl |  | H | 421.5 | 4.5 | 100 | 1 |  |
| **10** | N^10^-alkyl |   Full structure | - | 431.9 | 6.2 | 76 | 1 |  |
| **11** | N^10^-carbonyl |  | H | 420.4 | 4.6 | 8 | 1 | 0.106 |
| **12** | N^10^-methyl |   Full structure | - | 363.4 | 5.9 | 98 | 1 |  |
| **13** | N^10^ unsubstituted |   Full structure | - | 384.5 | 5.17 | 7 | 1 | 0.069 |
| **14** | N^10^ -sulfonyl |   Full structure | - | 369.5 | 2.6 | 68 | 1 |  |
| **15** | N^10^ unsubstituted |   Full structure | - | 474.4 | 5.7 | 4 | 1 | 0.068 |
| **16** | N^10^ unsubstituted |   Full structure | - | 489.4 | 6.9 | 16 | 1 | 0.398 |
| **17** | N^10^-**carbamoyl** |  | H | 480.6 | 3.8 | 21 | 1 | 0.631 |
| **18** | N^10^-carbonyl |  | H | 451.6 | 5.6 | 96 | 1 |  |
| **19** | N^10^-carbonyl |  | H | 408.3 | 5.6 | 100 | 1 |  |
| **20** | N^10^-alkyl |   Full structure | H | 413.9 | 6.9 | 100 | 1 |  |
| **21** | N^10^-carbonyl |  | H | 419.4 | 3.4 | 100 | 0.4 |  |
| **22^a^** | - | - | - |  |  | - | - | - |
| **23^a^** | - | - | - |  |  | - | - | - |
| **24** | N^10^-alkyl |   Full structure | H | 491.6 | 5.8 | 97 | 0.4 |  |
| **25** | N^10^-carbonyl |  | CF3 | 458.5 | 5.9 | 100 | 0.4 |  |
| **26**  DT-PTZ-C | **N^10^-carbamoyl** |  | H | 337.4 | 2.0 | 0 | 0.4 | 0.017 |
| **27** | **-** | - | - |  |  | - | - | 42 |
| **29** | **N^10^-aryl** |   Full structure | Cl | 500.4 | 10.2 | 100 | 0.4 |  |
| **30** | Fused **N^10^- carbonyl** |   Full structure | - | 357.7 | 4.4 | 90 | 0.4 |  |
| **31** | N^10^ unsubstituted |   Full structure |  | 456.6 | 6.4 | 17 | 1 | 0.055 |
| **32^a^** | - | - | - |  |  | - | - | - |
| **33** | N^10^-carbonyl |  | H | 352.4 | 1.9 | 14 | 1 | 0.646 |
| **34** | N^10^-carbonyl |  | H | 353.4 | 1.5 | 100 | 1 |  |
| **35** | N^10^-carbonyl |  | Cl | 469.9 | 4.6 | 100 | 1 |  |
| **36** | N^10^-carbonyl |  | H | 338.3 | 4.5 | 100 | 1 |  |
| **37** | **N^10^-carbamoyl** |  | H | 388.5 | 4.5 | 100 | 1 |  |
| **38** | **N^10^-carbamoyl** |  | H | 321.4 | 5.9 | 100 | 1 |  |
| **39^a^** | - | - | - |  |  | - | - | - |
| **40** | N^10^-carbonyl |  | CF3 | 408.4 | 2.8 | 0 | 1 | 0.042 |
| **41** | N^10^-carbonyl |  | H | 390.5 | 5.0 | 96 | 1 |  |
| **42** | N^10^-carbonyl |  | CF3 | 403.4 | 4.2 | 87 | 1 |  |
| **43** | N^10^-carbonyl |  | H | 335.4 | 3.2 | 83 | 1 |  |
| **44** | N^10^-carbonyl |  | CF3 | 450.3 | 7.4 | 85 | 1 |  |
| **45** | N^10^-carbonyl |  | CF3 | 421.5 | 4.0 | 84 | 1 |  |
| **46** | N^10^-carbonyl |  | H | 353.5 | 3.0 | 81 | 1 |  |
| **47** | N^10^-carbonyl |  | H | 421.4 | 2.5 | 75 | 1 |  |
| **48** | N^10^-carbonyl |  | CF3 | 447.5 | 4.7 | 77 | 1 |  |
| **49** | N^10^-carbonyl |  | H | 379.5 | 3.7 | 90 | 1 |  |
| **50** | N^10^-carbonyl |  | H | 457.6 | 3.1 | 89 | 1 |  |
| **51** | N^10^-carbonyl |  | H | 372.5 | 2.2 | 91 | 1 |  |
| **52** | N^10^-carbonyl |  | CF3 | 440.5 | 3.2 | 93 | 1 |  |
| **53** | **N^10^-carbamoyl** |  | H | 390.5 | 4.3 | 0 | 1 | 0.13 |
| **54** | N^10^-carbonyl |  | H | 354.4 | 2.3 | 100 | 1 |  |
| **55** | N^10^-carbonyl |  | H | 503.6 | 4.2 | 91 | 1 |  |
| **56** | N^10^-carbonyl |  | H | 417.5 | 3.4 | 86 | 1 |  |
| **57** | N^10^-carbonyl |  | H | 304.4 | 4.3 | 90 | 1 |  |
| **58** | N^10^-carbonyl |  | H | 304.4 | 4.3 | 85 | 1 |  |
| **59** | N^10^-carbonyl |  | H | 350.5 | 3.6 | 88 | 1 |  |
| **60** | N^10^-carbonyl |  | H | 309.4 | 5.2 | 89 | 1 |  |
| **61** | N^10^-carbonyl |  | Cl | 374.9 | 2.6 | 0 | 1 | 0.023 |
